# Neuroprotective peptide ADNF-9 in fetal brain of C57BL/6 mice exposed prenatally to alcohol

**DOI:** 10.1186/1423-0127-18-77

**Published:** 2011-10-21

**Authors:** Youssef Sari, Zaneer M Segu, Ahmed YoussefAgha, Jonathan A Karty, Dragan Isailovic

**Affiliations:** 1 Department of Pharmacology, College of Pharmacy and Pharmaceutical Sciences, University of Toledo, Toledo, OH; 2 Department of Chemistry, Indiana University, Bloomington, IN; 3 Department of Applied Health Science, Indiana University, Bloomington, IN; 4 Department of Chemistry, University of Toledo, Toledo, OH

## Abstract

**Background:**

A derived peptide from activity-dependent neurotrophic factor (ADNF-9) has been shown to be neuroprotective in the fetal alcohol exposure model. We investigated the neuroprotective effects of ADNF-9 against alcohol-induced apoptosis using TUNEL staining. We further characterize in this study the proteomic architecture underlying the role of ADNF-9 against ethanol teratogenesis during early fetal brain development using liquid chromatography in conjunction with tandem mass spectrometry (LC-MS/MS).

**Methods:**

Pregnant C57BL/6 mice were exposed from embryonic days 7-13 (E7-E13) to a 25% ethanol-derived calorie [25% EDC, Alcohol (ALC)] diet, a 25% EDC diet simultaneously administered i.p. ADNF-9 (ALC/ADNF-9), or a pair-fed (PF) liquid diet. At E13, fetal brains were collected from 5 dams from each group, weighed, and frozen for LC-MS/MS procedure. Other fetal brains were fixed for TUNEL staining.

**Results:**

Administration of ADNF-9 prevented alcohol-induced reduction in fetal brain weight and alcohol-induced increases in cell death. Moreover, individual fetal brains were analyzed by LC-MS/MS. Statistical differences in the amounts of proteins between the ALC and ALC/ADNF-9 groups resulted in a distinct data-clustering. Significant upregulation of several important proteins involved in brain development were found in the ALC/ADNF-9 group as compared to the ALC group.

**Conclusion:**

These findings provide information on potential mechanisms underlying the neuroprotective effects of ADNF-9 in the fetal alcohol exposure model.

## Background

Fetal alcohol exposure (FAE) or fetal alcohol syndrome (FAS) is a significant worldwide problem. Clinical studies demonstrate that brain growth deficits and neurological disorders are one of the pathological features of FAS or FAE [[1-4]; for review see Ref. [5]]. Experimental studies demonstrated that prenatal alcohol exposure induces brain growth restriction, microcephaly, facial dysmorphology, and abnormal behaviors [6-10].

Studies performed in our laboratory reveal that prenatal alcohol exposure induces brain growth deficits at different embryonic stages [for review see Ref. [11]]. The effects of prenatal alcohol exposure might be associated with an apoptotic mechanism [12]. This apoptotic mechanism involves intrinsic mitochondrial and extrinsic pathways such as receptor systems [13,14]. We have recently shown that prenatal alcohol exposure induced apoptosis that might be associated with activation of caspase-3, increases of cytosolic cytochrome c, and decreases of mitochondrial cytochrome *c *[15,16].

Label-free quantitative proteomic analyses using liquid chromatography in conjunction with a tandem mass spectrometry (LC-MS/MS) system showed significant alteration of mitochondrial, cytosolic, nuclear and cytoskeletal proteins in fetal brains exposed prenatally to alcohol [17]. Less is known about the treatment or prevention of the effects of prenatal alcohol exposure. Studies performed by us and others have shown potential preventive effects of prenatal alcohol exposure using derived peptides in animal models [11,15,16,18-20] and *in vitro *[20-23]. Among these peptides, SALLRSIPA, known as SAL or ADNF-9, is derived from activity dependent neurotrophic factor (ADNF) [24,25] and NAPVSIPQ peptide, termed NAP, is derived from activity-dependent neuroprotective protein (ADNP) [26,27]. In this study, we used histological assay (TUNEL staining) for determination of apoptosis and an LC-MS/MS system to investigate the proteins involved in ADNF-9 neuroprotection. We hypothesized that ADNF-9 administered alongside prenatal alcohol exposure can prevent alcohol-induced growth deficit and apoptosis through several key proteins that are involved in fetal brain development.

## Methods

### Animals

C57BL/6 mice were tested in this study. C57Bl/6 is an established and well studied model in the field of FAE and FAS [11,15-17,19,28,29]. These mice were supplied by Harlan, Inc. (Indianapolis, IN, USA). They were housed at the Indiana University Laboratory Animal Research Center in a vivarium with a controlled climate (temperature 22°C, and 30% humidity) and a 12:12 reverse light-dark cycle. Pregnant mice had free access to a liquid diet for 24 hours during the treatment period. All animal procedures were approved by the Institutional Animal Care and Use Committee of Indiana University Bloomington and are in accordance with the guidelines of the Institutional Animal Care and Use Committee at the National Institutes of Health and the Guide for the Care and Use of Laboratory Animals. Note that this study was performed in part at Indiana University and The University of Toledo. Animal treatments alongside exposure to liquid diet were performed at Indiana University Bloomington. TUNEL staining and proteomics were also performed at Indiana University. Additional TUNEL staining and cell count were performed at the University of Toledo.

### Breeding and treatments

Female mice were placed in the male home cage for 2 hours. Females were then checked for a sperm plug by vaginal smear. E0 was designated as the time point when the vaginal smear was positive. Weight-matched pregnant females were assigned on E7 to the following groups: (1) Ethanol liquid diet group (ALC, n = 5), which was fed with chocolate sustacal (supplemented with vitamins and minerals); liquid diet 25% (4.49%, v/v) ethanol-derived calories (EDC); (2) pair-fed control groups (PF to ethanol-fed group, n = 5), which was fed with a maltose-dextrin solution isocaloric to the dose of ethanol used; and (3) treatment group, which received ADNF-9 i.p. injection alongside alcohol exposure in liquid diet (ALC/ADNF-9, 30 μg/20 g of body weight, n = 5). The PF group dam, yoked individually to an ALC dam, was given daily amounts of matched isocaloric liquid diet with maltose-dextrin substituted for ethanol at all times during gestation (E7-E13). PF animals were yoked to ALC or ALC/ADNF-9 animals. The amounts of liquid diet and body weight of the dams were controlled and not different between all groups. Pregnant mice had continuous, 24-hour free access to the alcohol liquid diet or PF liquid diet for 7 days. All groups were exposed to free choice liquid diet drinking and no solid food was provided.

We used the fortified liquid diet that contained 237 ml of chocolate-flavored sustacal, 1.44 g vitamin diet fortification mixture, and 1.2 g salt mixture XIV [30,31]. For the ethanol diet, 15.3 ml (4.49% v/v, 25% EDC) of 95% ethanol was mixed with the fortified chocolate-flavored sustacal, adjusted with water, to make 320 ml of diet with 1 cal/ml (ethanol). The isocaloric control diet was prepared by adding 20.2 g maltose-dextrin to the fortified chocolate-flavored sustacal with water to bring it to 1 cal/ml. A day prior to treatment, the ALC, PF, and ALC/ADNF-9 groups were adapted to the liquid diet. The body weights of the dams were recorded every day during the treatments. A consumed liquid diet during a 24-hour period was recorded from 30-ml graduated screw-cap tubes, and a freshly prepared diet was provided each day. The ALC and ALC/ADNF-9 groups had free access to the ethanol liquid diet delivering 25% EDCs as the sole source of nutrients.

### Animal and fetal brain extractions

Pregnant mice were euthanized by CO_2 _followed by cervical dislocation on E13, and the fetuses were removed. This method is consistent with the recommendations of the Panel on Euthanasia of the American Veterinary Medical Association. The fetal brains were further dissected, by an experimenter blind to the treatment groups, from the base of the primordium olfactory bulb to the base of the metencephalon. From the same dam, at least 5 fetal brains were randomly selected, weighed, frozen and stored at -70°C until used for proteomic assay and other fetal brains from each dam were postfixed in 4% paraformaldehyde for TUNEL assay.

### TUNEL assay for determination of cell death

Determination of cell death was performed using TUNEL reaction (TdT-mediated dUTP Nick End Labeling) as recently described in our studies [15-17]. Fetal brains from control and treated groups, fixed in 4% paraformaldehyde, were embedded as pairs in gelatin for immunostaining consistency. These fetal brains embedded in gelatin were sectioned at 50 μm thickness using a Leica vibratome apparatus (W. Nuhsbaum, Inc). Fetal brain sections were fixed in superfrost plus slides and then treated with Proteinase K (10-20 μg/ml) for 5 minutes at 37°C, rinsed with PBS three times for 5 minutes and then incubated with 3% H_2_O_2 _in methanol for 10 minutes at room temperature. The fetal brain sections were again rinsed with PBS three times for 5 minutes and then incubated in a permeabilisation solution (0.1% TX-100 in 0.1% sodium citrate) for 2 minutes at 4°C. After the fetal brain sections were rinsed twice in PBS for 5 minutes they were incubated with a TUNEL reaction mixture (50 μl from bottle 1 and 450 μl from bottle 2, Roche Pharmaceuticals, Inc, IN) for 1 hour at 37°C. The control was prepared by incubation of tissue sections only in solution from bottle 2. The sections were rinsed three times for 5 minutes with PBS and incubated in converter-POD for 30 minutes at 37°C. After the fetal brain sections were rinsed with TBS, they were incubated in 0.05% 3'-3'-diaminobenzidine tetrahydrochloride and 0.003% H_2_O_2 _in TBS to detect the activity of peroxidase. Fetal brain sections were Nissl-counterstained with 0.5% cresyl violet to determine the cellular profile and then dehydrated with ethanol. The slides were mounted with a permount mounting media for microscope observation and TUNEL-positive cell counts.

The number of TUNEL-positive cells was evaluated in the primordium cingulate cortex of fetal brains. Four sections collected from one fetal brain from one litter were counted for TUNEL-positive cells. We have counted the entire population of TUNEL-positive cells manually in every other section in the primordium cingulate cortex, and this was performed to overcome the bias of over-counting the TUNEL-positive cells. The data represented the average of all the counted sections.

### Protein extraction and trypsin digestion

Frozen fetal brain tissues were thawed and homogenized at 4°C in 50 mM (600 μL) ammonium bicarbonate using Tissue-Tearor™ homogenizer (BioSpec Products, Bartlesville, OK) by gradually increasing the speed to 30,000 rpm for 15 minutes. The extract was centrifuged at 14,000 rpm for 1 hour at 4°C; the supernatant containing proteins was collected for analysis. The total protein concentration of the sample was determined by Bradford protein assay (Bio Rad, Hercules, CA, USA). Proteins extracted from the supernatant were digested by trypsin for LC-MC/MS analysis.

Trypsin digestion assay was performed by initially adding 1% acid-labile surfactant (RapidGest Waters, Milford, MA, USA) and denaturing the extracted proteins for 5 minutes at 95°C. The extract was then incubated with 5 mM Dithiothreitol (DTT) at 60°C for 45 minutes. Alkylation was achieved by adding iodoacetamide (IAA) to a final concentration of 20 mM prior to incubation at room temperature for 45 minutes in the dark. A second aliquot of DTT was then added to the sample, bringing the final concentration of DTT to 10 mM. The samples were then incubated at room temperature for 30 minutes to quench the alkylation reaction. Trypsin was added (1:30 w/w), and the solutions were incubated at 37°C for 18 hours. The enzymatic digestion was finally quenched through an addition of formic acid.

### Instrumentation

LC-MS/MS analyses of the tryptic digests were performed using a Dionex 3000 Ultimate nano-LC system (Dionex, Sunnyvale, CA) interfaced to a LTQ Orbitrap hybrid mass spectrometer (Thermo Scientific, San Jose, CA). Prior to separation, a 2-μl aliquot of trypsin digestion (1 μg protein equivalent) was loaded isocratically with 3% acetonitrile and 0.1% formic acid onto a PepMap300 C18 cartridge (5 μm, 300 Å, Dionex) to purify the sample from salt and buffers. The peptides were then separated on a pulled-tip (New Objective, Woburn, MA) capillary column (150 mm × 75 μm i.d) packed with 3 μm and 120 Å pore-sized resin bonded with Aqua C18 (Phenomenex, Torrance, CA) using a reversed-phase gradient 3-55% of acetonitrile with 0.1% formic acid over 85 minutes for proteins extracted from fetal brain tissues, at 300 nl/min flow rate. The mass spectrometer was operated in an automated data-dependent mode switched between an MS scan and CID-MS. In this mode, eluted LC products undergo an initial full-spectrum MS scan from *m*/*z *300 to 2000 in the Orbitrap at 15,000 mass resolutions. Subsequently, CID-MS (at 35% normalized collision energy) was performed in the ion trap. The precursor ion was isolated using the data-dependent acquisition mode with a 2 *m*/*z *isolation width to select, automatically and sequentially, the five most intense ions (starting with the most intense) from the survey scan. The total cycle (6 scans) is continuously repeated for the entire LC-MS run under data-dependent conditions with dynamic exclusion set to 60 seconds. Performing MS scanning in the Orbitrap offers high mass accuracy and accurate charge state assignment of the selected precursor ions.

### Database searching and quantification

Mascot version 2.1.3 was used for all search results obtained in this work. The data were searched against the Swiss-Prot database for house mice. Trypsin was selected as the enzyme, and one missed cleavage was allowed. A carbomidomethyl was selected as a fixed modification of all cysteine residues, and acetyl (N-term) and oxidation (M) were selected as variable modifications. The mass tolerance of both MS and MS/MS data were set to 0.2 and 0.8 Da, respectively. Peptides with mass accuracy higher than 2 ppm, Mascot ion score of 30 and above, and proteins with 2 or more peptide matches were considered as positive identifications. The quantitative analysis of proteins was carried out using ProteinQuant Suite software developed at Indiana University [32]. Briefly, the raw data obtained from the LTQ-Orbitrap XL mass spectrometer were converted to MASCOT generic files (MGFs). MGFs were then parsed with ProtParser, subject to specific parsing criteria. The minimum MOWSE score was set to 30, and proteins with 2 or more peptide matches were considered a confident match. The peptide mass threshold, peak width and apex assignment windows were set to 600 Da. All parsed files were combined into a master file that contains the list of all proteins and peptides identified in the span of all the processed LC-MS/MS analyses. Then, the combined master files, incorporated with their corresponding mzXML files, were submitted to ProteinQuant as described previously [32].

### Data evaluation and analyses

Principal component analysis (PCA) was performed using MarkerView software (AB Sciex, Concord, Ontario, Canada). Unsupervised PCA was employed without using prior knowledge of the sample groups. MS data were weighted using logarithm function and scaled by pareto function, in which each value was subtracted from the average value and divided by the square root of the standard deviation. In this way, intense peaks were prevented from completely dominating the PCA, and any peaks with a good signal-to-noise ratio had more importance in the PCA. Dot plots were plotted using Origin software (OriginLab Corporation, Northampton, MA).

The range of values obtained in this study are expressed as a standard error of mean (S.E.M.). The comparisons of the levels of proteins reflecting the levels of proteins between ALC and ALC/ADNF-9 were performed using the Wilcoxon rank sum test [33], also known as the Mann-Whitney rank sum test. The p-values demonstrating statistically significant differences between ALC and ALC/ADNF-9 are reported in Table 1. All statistical analyses were performed using SAS, version 9.1.

**Table 1:** Proteins, among others, that have been significantly down-regulated or up-regulated in their expression as a consequence of administration of ADNF-9 against the effect of prenatal alcohol exposure in E13 fetal brains

Protein	Function	ALC group	ALC/ADNF-9 group	*p*-value
**Heterogeneous nuclear ribonucleoprotein U-like protein (HNRL2_MOUSE)**	Acts as a basic transcriptional regulator. Represses basic transcription driven by several cellular promoters. When associated with BRD7, activates transcription of glucocorticoid-responsive promoter in the absence of ligand-stimulation. Plays also a role in mRNA processing and transport. Binds avidly to poly(G) and poly(C) RNA homopolymers in vitro.	5.7E-05 ± 8.02E-06	8.1E-05 ± 2.91E-06	0.021

**Dynein light chain 2, cytoplasmic (DYL2_MOUSE)**	Acts as one of several non-catalytic accessory components of the cytoplasmic dynein 1 complex that are thought to be involved in linking dynein to cargos and to adapter proteins that regulate dynein function. Cytoplasmic dynein 1 acts as a motor for the intracellular retrograde motility of vesicles and organelles along microtubules.	7.1E-04 ± 5.13E-05	8.8E-04 ± 3.71E-05	0.036

**Hemoglobin subunit epsilon-Y2 (HBE_MOUSE)**	Hemoglobin epsilon chain is a beta-type chain found in early embryos.	1.4E-02 ± 4.88E-04	2.1E-02 ± 3.18E-03	0.021

**Cyclin-dependent kinase inhibitor 1B (CDN1B_MOUSE)**	Important regulator of cell cycle progression. Involved in G1 arrest. Potent inhibitor of cyclin E- and cyclin A-CDK2 complexes. Positive regulator of cyclin D-dependent kinases such as CDK4. Regulated by phosphorylation and degradation events.	1.2E-05 ± 9.23E-07	1.8E-05 ± 1.31E-06	0.012

**Peptidyl-prolyl cis-trans isomerase FKBP4 (FKBP4_MOUSE)**	Immunophilin protein with PPIase and co-chaperone activities. Component of unliganded steroid receptors heterocomplexes through interaction with heat-shock protein 90 (HSP90). May play a role in the intracellular trafficking of heterooligomeric forms of steroid hormone receptors between cytoplasm and nuclear compartments. The isomerase activity controls neuronal growth cones via regulation of TRPC1 channel opening. Acts also as a regulator of microtubule dynamics by inhibiting MAPT/TAU ability to promote microtubule assembly.	8.6E-04 ± 7.35E-05	1.1E-03 ± 4.39E-05	0.036

**RNA-binding protein Raly (RALY_MOUSE)**	Probable-RNA binding protein. Could be a heterogeneous nuclear ribonucleoprotein (hnRNP). May be involved in pre-mRNA splicing.	9.2E-05 ± 8.44E-06	1.3E-04 ± 9.82E-06	0.012

**60S ribosomal protein L12 (RL12_MOUSE)**	Binds directly to 26S ribosomal RNA.	2.3E-03 ± 9.60E-05	3.0E-03 ± 1.06E-04	0.012

**Splicing factor 3B subunit 3 (SF3B3_MOUSE)**	Subunit of the splicing factor SF3B required for 'A' complex assembly formed by the stable binding of U2 snRNP to the branchpoint sequence (BPS) in pre-mRNA. Sequence independent binding of SF3A/SF3B complex upstream of the branch site is essential; it may anchor U2 snRNP to the pre-mRNA. May also be involved in the assembly of the 'E' complex. Belongs also to the minor U12-dependent spliceosome, which is involved in the splicing ofa rare class of nuclear pre-mRNA intron.	5.0E-04 ± 2.52E-05	6.5E-04 ± 5.20E-05	0.036

**Peroxiredoxin-2 (PRDX2_MOUSE)**	Involved in redox regulation of the cell. Reduces peroxides with reducing equivalents provided through the thioredoxin system. It is not able to receive electrons from glutaredoxin. May play an important role in eliminating peroxides generated during metabolism. Might participate in the signaling cascades of growth factors and tumor necrosis factor-alpha by regulating the intracellular concentrations of H_2_O_2_.	3.1E-03 ± 2.60E-04	2.3E-03 ± 1.84E-04	0.036

**Serine/threonine-protein phosphatase PP1-beta catalytic subunit (PP1B_MOUSE)**	Protein phosphatase (PP1) is essential for cell division; it participates in the regulation of glycogen metabolism, muscle contractility and protein synthesis. Involved in regulation of ionic conductances and long-term synaptic plasticity.	3.7E-04 ± 3.68E-05	5.1E-04 ± 4.32E-05	0.036

**Endoplasmin (ENPL_MOUSE)**	Molecular chaperone that functions in the processing and transport of secreted proteins. Functions in endoplasmic reticulum associated degradation (ERAD). Has ATPase activity.	5.3E-03 ± 4.89E-04	6.8E-03 ± 2.83E-04	0.036

**Dihydropyrimidinase-related protein 1 (DPYL1_MOUSE)**	Necessary for signaling by class 3 semaphorins and subsequent remodeling of the cytoskeleton. Plays a role in axon guidance, invasive growth and cell migration.	3.2E-03 ± 9.22E-05	3.7E-03 ± 1.06E-04	0.012

**Serine/arginine-rich splicing factor 3 (SFRS3_MOUSE)**	May be involved in RNA processing in relation with cellular proliferation and/or maturation.	7.6E-04 ± 7.33E-05	1.0E-03 ± 3.86E-05	0.036

**Heat shock protein HSP 90-alpha (HS90A_MOUSE)**	Molecular chaperone. Has ATPase activity	6.1E-03 ± 2.95E-04	7.1E-03 ± 2.08E-04	0.036

**Hemoglobin subunit beta-1 (HBB1_MOUSE)**	Involved in oxygen transport from the lung to the various peripheral tissues.	2.1E-03 ± 1.05E-04	2.8E-03 ± 1.04E-04	0.012

**Transketolase (TKT_MOUSE)**	Transketolase: A transferase bringing about the reversible interconversion of sedoheptulose 7-phosphate and d-glyceraldehyde 3-phosphate to produce d-ribose 5-phosphate and d-xylulose 5-phosphate, and also other similar reactions, such as hydroxypyruvate and an aldehyde into CO2 and an extended hydroxypyruvate; a part of the nonoxidative phase of the pentose phosphate pathway.	2.4E-03 ± 1.21E-04	1.6E-03 ± 1.34E-04	0.012

**Casein kinase II subunit beta (CSK2B_MOUSE)**	Plays a complex role in regulating the basal catalytic activity of the alpha subunit. Participates in Wnt signaling.	4.0E-05 ± 4.24E-06	5.5E-05 ± 3.36E-06	0.021

**Microtubule-associated protein 1B (MAP1B_MOUSE)**	The function of brain MAPS is essentially unknown. Phosphorylated MAP1B may play a role in the cytoskeletal changes that accompany neurite extension. Possibly MAP1B binds to at least two tubulin subunits in the polymer, and this bridging of subunits might be involved in nucleating microtubule polymerization and in stabilizing microtubules.	1.3E-03 ± 5.87E-05	1.6E-03 ± 1.31E-04	0.036

**Hemoglobin subunit zeta (HBAZ_MOUSE)**	The zeta chain is an alpha-type chain of mammalian embryonic hemoglobin, synthesized primarily in the yolk sac.	4.1E-03 ± 2.76E-04	5.2E-03 ± 3.18E-04	0.036

**Eukaryotic translation initiation factor 5A-1 (IF5A1_MOUSE)**	mRNA-binding protein involved in translation elongation. Has an important function at the level of mRNA turnover, probably acting downstream of decapping. Involved in actin dynamics and cell cycle progression, mRNA decay and probably in a pathway involved in stress response and maintenance of cell wall integrity. With syntenin SDCBP, functions as a regulator of TP53/p53 and TP53/p53-dependent apoptosis. Also regulates TNF-alpha-mediated apoptosis. Mediates effects of polyamines on neuronal process extension and survival. May play an important role in brain development and function and in skeletal muscle stem cell differentiation.	4.2E-03 ± 2.03E-04	5.3E-03 ± 3.68E-04	0.036

**Fatty acid synthase (FAS_MOUSE)**	Fatty acid synthetase catalyzes the formation of long-chain fatty acids from acetyl-CoA, malonyl-CoA and NADPH. This multifunctional protein has 7 catalytic activities and an acyl carrier protein.	1.8E-03 ± 1.12E-04	2.1E-03 ± 8.41E-05	0.036

**Histone-binding protein RBBP4 (RBBP4_MOUSE)**	Core histone-binding subunit that may target chromatin assembly factors, chromatin remodeling factors and histone deacetylases to their histone substrates in a manner that is regulated by nucleosomal DNA. Component of several complexes that regulate chromatin metabolism. These include the chromatin assembly factor 1 (CAF-1) complex, which is required for chromatin assembly following DNA replication and DNA repair, and the core histone deacetylase (HDAC) complex, which promotes histone deacetylation and consequent transcriptional repression.	6.6E-04 ± 4.48E-05	8.2E-04 ± 2.98E-05	0.036

**Nuclear cap-binding protein subunit 1 (NCBP1_MOUSE)**	Component of the cap-binding complex (CBC), which binds co-transcriptionally to the 5' cap of pre-mRNAs and is involved in various processes such as pre-mRNA splicing, translation regulation, nonsense-mediated mRNA decay, RNA-mediated gene silencing (RNAi) by microRNAs (miRNAs) and mRNA export. The CBC complex is involved in mRNA export from the nucleus via its interaction with THOC4/ALY, leading to the recruitment of the mRNA export machinery to the 5' end of mRNA and to mRNA export in a 5' to 3' direction through the nuclear pore.	4.6E-05 ± 7.84E-06	7.7E-05 ± 4.31E-06	0.021

Values are expressed as protein areas and their S.E.M.

Statistical analyses of the number of TUNEL-positive cells and fetal brain weights were performed using one-way analysis of variance (ANOVA) and Newman-Keuls multiple comparison test between the PF, ALC, and ALC/ADNF-9 groups. All tests of significance were set at p < 0.05.

## Results

### Fetal brain weight

**Figure 1:**
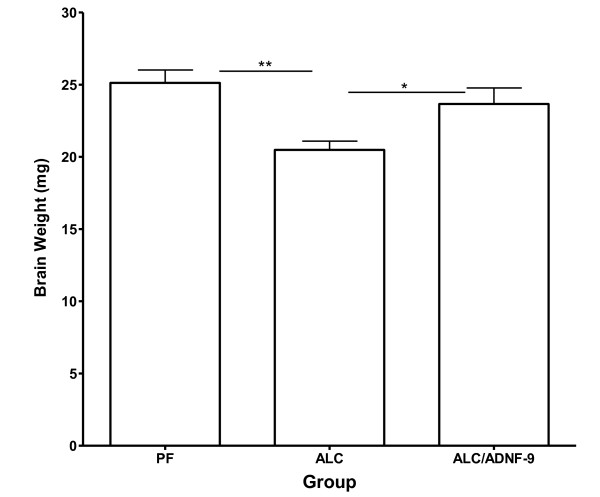
**Neuroprotective effect of ADNF-9 in fetal brains exposed prenatally to alcohol at E13**. Prenatal alcohol exposure induced significant reduction in fetal brain weight in the ALC group as compared to the PF group (p < 0.01). ADNF-9 administration alongside prenatal alcohol exposure prevented alcohol-induced reduction in fetal brains weights (p < 0.05). Values are expressed as means ± SEM. N = 5 for each group. *p < 0.05, **p < 0.01 (Newman-Keul's post hoc test).

Fetal brain weights from each litter were averaged and the averaged value was used as one number (n). Statistical analyses of fetal brain weights demonstrate a significant weight reduction in the ALC group as compared to the PF control group (Figure 1, p < 0.01). Importantly, treatment of pregnant mice with ADNF-9 alongside alcohol exposure shows a preventive effect against alcohol-induced reduction in fetal brain weight. Statistical analyses show significant differences between the ALC/ADNF-9 and ALC groups (Figure 1, p < 0.05). There was no significant difference in fetal brain weights between the ALC/ADNF-9 and PF groups.

### TUNEL staining identifying cell death

**Figure 2:**
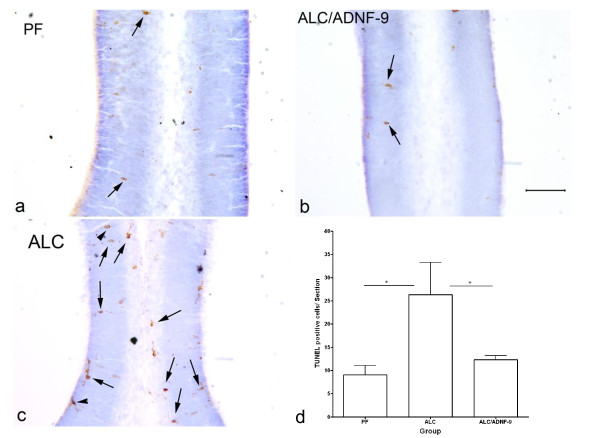
**Neuroprotective effect of ADNF-9 against alcohol-induced cell death in primordium cingulate cortex at E13**. Prenatal alcohol exposure induced increases in TUNEL-positive cells. Importantly, administration of ADNF-9 prevented the alcohol-induced increases in cell death (a-c). Note that cells undergoing apoptosis are indicated by cell processes as shown by arrowheads. However, arrows indicate cells in the final stage of apoptosis. Statistical analyses demonstrate a significant difference between groups (p = 0.0405). (d) Prenatal alcohol exposure induced significant increases in the number of TUNEL-positive cells in the ALC group as compared to the PF (p < 0.05). ADNF-9 administration prevented significantly the alcohol-induced increases in the number of TUNEL-positive cells (p < 0.05). Values are expressed as means ± SEM. N = 4 for each group. *p < 0.05 (Newman-Keul's post hoc test). Scale bar = 100 μm.

TUNEL staining was used to determine cell death. We tested ADNF-9 to investigate its neuroprotective effect against alcohol-induced apoptosis. We have focused our anatomical and statistical analysis in one area of the fetal brains, which is the primordium cingulate cortex. This fetal brain region has been well studied in previous work [11,15]. Anatomical observation shows an increase in TUNEL-positive cells in the ALC group (Figure 2c) as compared to the PF (Figure 2a) and ALC/ADNF-9 (Figure 2b) groups. Statistical analyses of the cell counts reveal a significant reduction in the number of TUNEL-positive cells in the ALC group as compared to the PF control group (p < 0.05) (Figure 2d). Treatment with ADNF-9 alongside prenatal alcohol exposure prevented alcohol-induced increases in the number of TUNEL-positive cells as compared to the ALC group (p < 0.05).

### LC-MS/MS protein analyses

LC-MS/MS analyses of the extracted proteomes from each group resulted in the identification of 598 proteins. As performed in a recent study [17], the peptide identification was performed using the MASCOT search engine and a filtering criteria that resulted in at least a 95% identification confidence and a false-positive identification rate < 5%. The information related to the functionality of the identified proteins were obtained from the Swiss-Model Repository http://swissmodel.expasy.org/ and UniProtKB http://www.uniprot.org/.

### Protein identifications using LC-MS/MS quantitative analyses

**Figure 3:**
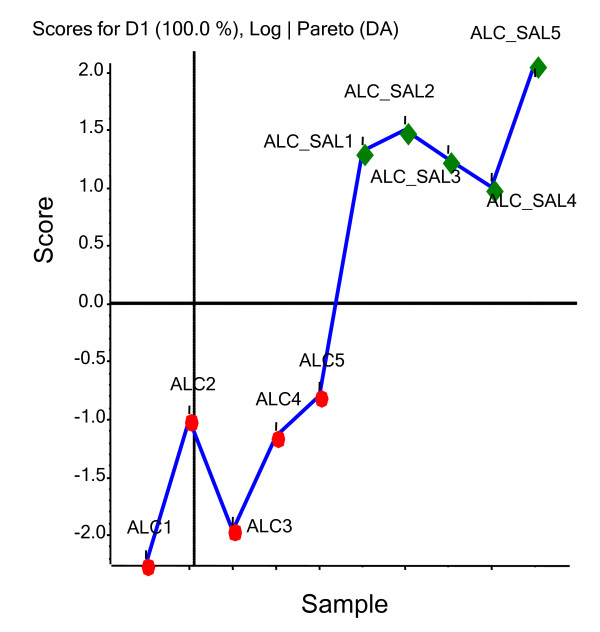
**PCA score plot of the levels of the identified proteins for the analyzed groups: ALC and ALC/SAL(ADNF-9)**.

PCA score plots of the levels of all identified proteins between the ALC and ALC/SAL(ADNF-9) groups are shown in Figure 3. Differences in the levels of proteins between the ALC and ALC/SAL(ADNF-9) groups show distinct clusters. Table 1 displays proteins that are significantly different and contributed to the distinct clusters observed in Figure 3.

**Figure 4:**
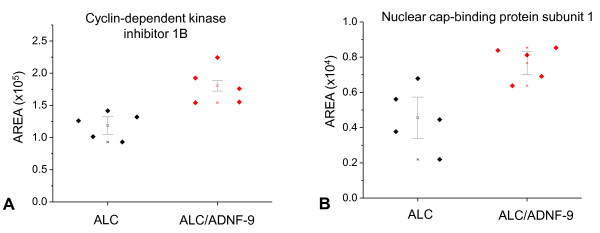
**Proteins that are significantly upregulated in the ALC/ADNF-9 group as compared to the ALC group, cyclin-dependent kinase inhibitor 1B (a), and nuclear cap-binding protein subunit 1 (b)**.

We have focused our proteomic analyses on both the ALC and ALC/SAL(ADNF-9) groups in order to determine the effects of ADNF-9 administration in the changes of the level of expression of proteins. Table 1 shows all the proteins that are regulated as a result of ADNF-9 administration alongside prenatal alcohol exposure. Administration of ADNF-9 alongside prenatal alcohol exposure upregulates key proteins involved in cell cycle progression and cell division including cyclin-dependent kinase inhibitor 1B (p = 0.012) (Figure 4a) and serine/threonine-protein phosphatase PP1-beta catalytic subunit (p = 0.036) in the ALC/ADNF-9 group as compared to the ALC group (Table 1). ADNF-9 administration also prevented alcohol-induced reduction in the level of expression of proteins involved in axon guidance and cellular proliferation such as dihydropyrimidinase-related protein 1 (p = 0.012) and serine/arginine-rich splicing factor 3 in the ALC/ADNF-9 group as compared to the ALC group (Table 1). In addition, administration of ADNF-9 alongside prenatal alcohol exposure upregulates some proteins involved in microtubule organization and function; these proteins include peptidyl-prolyl cis-trans isomerase (p = 0.036), microtubule-associated protein 1B (p = 0.036) and dynein light chain 2 (p = 0.036) (Table 1). Moreover, ADNF-9 administration alongside prenatal alcohol exposure upregulates some nuclear proteins involved in gene transcription such as RNA-binding protein Raly (p = 0.012) (Table 1), eukaryotic translation initiation factor 5A-1 (p = 0.028) (Table 1), nuclear cap-binding protein subunit 1 (p = 0.016) (Figure 4B), and histone-binding protein RBBP4 (p = 0.02828) (Table 1) in the ALC/ADNF-9 group as compared to the ALC group.

## Discussion

We report here that alcohol exposure during pregnancy resulted in downregulation of fetal brain weights and increased in TUNEL-positive cells at E13 age. Importantly, ADNF-9 administration alongside prenatal alcohol exposure prevented alcohol-induced decreases in fetal brain weights and increases in cell death at E13. We chose to expose the pregnant mice from E7 to E13 based on studies indicating that the developing brain exhibited the highest susceptibility to alcohol exposure between E7 and later embryonic stages [29]. Using a similar drinking paradigm to these studies, we previously demonstrated that prenatal alcohol exposure from E7 to E13, E15 and E8 induced reduction in fetal brain weights and in the number of serotonin neurons, alteration of neurotransmitters, and induced neural tube defects [11,15,16,28,34]. In this study, we revealed that the neurotrophic peptide, ADNF-9, prevents the reduction in fetal brains that might be associated with the prevention of cell death or apoptosis in the primordium cingulate cortex. Previous studies have shown that prenatal alcohol exposure induced alterations in several fetal brain regions, including primordium cerebral cortex, ganglionic eminence, primordium thalamus, and primodrium septum [4,5,11,35]. It is noteworthy that alterations of the organization of primordium cortices by alcohol exposure might be associated with deficits in learning, memory, motor skills, and visual-spatial skills found in children born from mothers with habits of heavy drinks of alcohol during pregnancy [4,36,37].

On the other hand, we used LC-MS/MS to determine the differential protein expressions between ALC and ALC/ADNF-9 treated groups. Using LC-MS/MS, we recently showed that prenatal alcohol exposure induced alteration in mitochondrial, cytosolic and nuclear proteins in ALC as compared to PF control group [17]. Here, we focused our study to investigate the role of trophic peptide, ADNF-9, in prevention of alcohol-induced alteration of key proteins that are involved in fetal brain development. Thus, quantitative proteomic analyses revealed differential expression of proteins involved in cell cycle division and neuronal growth at E13. Among proteins upregulated in the ALC/ADNF-9 group as compared to the ALC group are cyclin-dependent kinase inhibitor 1B (CDN1B_MOUSE), serine/threonine-protein phosphatase PP1-beta catalytic subunit (PP1B_MOUSE), and dihydropyrimidinase-related protein 1 (DPYL1_MOUSE). Cyclin-dependent kinase inhibitor is an important regulator of cell cycle progression. This is in accordance with previous evidence indicating that prenatal alcohol exposure induced downregulation of cyclin-dependent kinase inhibitor and cyclin-dependent kinases [38]. It is also reported that prenatal alcohol exposure has been shown to delay cell cycle [39]. Moreover, *in vitro *study reveals that alcohol exposure alters the cell cycle regulatory factors [40]. Upregulation of cyclin-dependent kinase inhibitor as a consequence of ADNF-9 administration is an indication of the preventive effect against alcohol-induced alteration in cell cycle progression. It is possible that upregulation of cyclin-dependent kinase might be mediated through indirect action of ADNF-9. Indeed, the indirect upregulatory action of ADNF-9 in cyclin-dependent kinase might be associated with ADNF-9 neuroprotection, which consequently can prevent the alteration of cell cycle division. Moreover, ADNF-9 administration overcomes the downregulation of serine/threonine-protein phosphatase, which is involved in protein synthesis that is essential for cell division. It is unknown about the mechanisms of action of ADNF-9 involving these cell cycle proteins. Studies are warranted to investigate these mechanisms of action.

On the other hand, dihydropyrimidinase-related protein 1, a protein that plays a role in axon guidance, invasive growth and cell migration, was found upregulated in the ALC/ADNF-9 group. This protein also has a role in the remodeling of the cytoskeleton. Another protein from the same family was also found downregulated in the ALC group, as reported recently [17]. It is noteworthy that prenatal alcohol exposure altered brain growth and retarded the migration of neurons [for review see Ref. [11]]. Thus, ADNF-9 administration might prevent these deficits found in the FAE model.

Differential expression of proteins involved in transcription and gene function for cellular growth are identified at E13. Among the proteins upregulated in the ALC/ADNF-9 group, as compared to the ALC group, are heterogeneous nuclear ribonucleoprotein U-like protein (HNRL2_MOUSE), RNA-binding protein Raly (RALY_MOUSE), splicing factor 3B subunit 3 (SF3B3_MOUSE), serine/arginine-rich splicing factor 3 (SFRS3_MOUSE), eukaryotic translation initiation factor 5A-1 (IF5A1_MOUSE), histone-binding protein RBBP4 (RBBP4_MOUSE), and nuclear cap-binding protein subunit (NCBP1_MOUSE). In this study, we found that ADNF-9 administration induced upregulation of major nuclear proteins that are involved in the regulatory function of the transcription factors. Heterogeneous nuclear ribonucleoprotein acts as a basic transcriptional regulator that represses basic transcription, which might be driven by several cellular promoters. RNA-binding protein Raly is involved in pre-mRNA splicing. The splicing factor 3B subunit 3, found upregulated in the ALC/ADNF-9 group, is a subunit of the splicing factor SF3B required for complex assembly formed by the stable binding of U2 snRNP to the branchpoint sequence in pre-mRNA. In addition, ADNF-9 upregulates the nuclear cap-binding protein subunit; involves pre-mRNA splicing and translation regulation. On the other hand, ADNF-9 administration upregulates serine/arginine-rich splicing factor 3, which is involved in RNA processing associated with cellular proliferation and maturation. It has been demonstrated that prenatal alcohol exposure reduced cell proliferation [41]. Thus, ADNF-9 may have prevented alcohol-induction of this deficit through the splicing factor 3. ADNF-9 neuroprotection involves also a eukaryotic translation initiation factor, which is associated with actin dynamics and cell cycle progression for maintaining cell integrity. Studies are warranted to determine whether ADNF-9 is directly or indirectly associated with these identified proteins in the prevention of alcohol-induced apoptosis.

Upregulation of the level of histone-binding protein RBBP4 was found in the ALC/ADNF-9 treated group. This protein is considered as a core histone-binding subunit that interacts with chromatin assembly proteins, chromatin remodeling factors and histone deacetylases to their histone substrates. Alcohol exposure is known to disrupt histone and histone-binding proteins, which together can lead to epigenetic imprinting. This phenomenon is currently considered a major problem in FAE. The mechanisms of action involving the epigenetic imprinting are mainly DNA methylation and histone modifications (acetylation, methylation, and phosphorylation) that regulate gene transcription [42-46]. Covalent histone modifications via acetylation and deacetylation are key players in the changes in chromatin structure that consequently regulate gene expression [43,44,46].

Quantitative proteomic analyses demonstrated differential expression of proteins involved in cytoskeletal machinery. Among these proteins are dynein light chain 2 (DYL2_MOUSE), peptidyl-prolyl cis-trans isomerase FKBP4 (FKBP4_MOUSE), and microtubule-associated protein 1B (MAP1B_MOUSE). MAP1B, belonging to a microtubule-associated protein family, is a major cytoskeletal protein located in axonal as well as dendritic neuronal processes [47]. Recent studies reveal that chronic ethanol exposure alters the expression, assembly and cellular organization of the cytoskeleton, including actin and microtubules in vitro culture of hippocampus neurons [48]. Upregulation of MAP1B in the ALC/ADNF-9 group overcomes these alterations. *In vivo *and *in vitro *studies performed by us and others show that microtubule-associate protein 2 (MAP2) was also found to be downregulated in the ALC group as compared to the control group [17,49]. Moreover, DYL2 is a protein that acts as a motor protein for the intracellular retrograde motility of vesicles and organelles along microtubules. Upregulation of this protein in the ALC/ADNF-9 group prevents the alteration of intracellular retrograde trafficking. The peptidyl-prolyl cis-trans isomerase is an enzyme that controls neuronal growth cones by acting as a regulator of microtubule dynamics. It is noteworthy that ADNF-9 administration alongside ALC exposure prevents the alteration of key proteins involved in cytoskeletal protein function to maintain normal neuronal growth.

## Conclusions

ADNF-9 administration alongside prenatal alcohol exposure prevented alcohol-induced reduction in fetal brain weights and alcohol-induced increases in TUNEL-positive cells. Quantitative proteomic analyses were used in this study to determine differential proteins involved in ADNF-9 neuroprotection in fetal brains exposed prenatally to alcohol. We have identified several target proteins that were upregulated through ADNF-9 administration in the FAE model. Among these proteins are the proteins involved in cell division and cell growth, nuclear and/or transcriptional proteins, and cytoskeletal proteins. The mechanisms of action of ADNF-9 neuroprotection against alcohol-induced apoptosis might be mediated directly or indirectly through these identified proteins. These findings suggest that ADNF-9 might be used as a compound for the treatment against the effects of alcohol exposure during gestation.

## Competing interests

The authors declare that they have no competing interests.

## Authors' contributions

YS designed and conceptualized the study, interpretation of data related to TUNEL assay and proteomics, and wrote the manuscript. ZMS performed proteomics assay, generated the data and participated in writing the section dealing with methods of proteomics. AY performed the statistical analyses of all the proteomics data. JAK supervised the proteomics assay. DI performed principal component analysis and plotted dot plots. All authors read and approved the final version of the manuscript.
